# Association between epicardial adipose tissue and incident heart failure mediating by alteration of natriuretic peptide and myocardial strain

**DOI:** 10.1186/s12916-023-02836-4

**Published:** 2023-03-29

**Authors:** Manting Choy, Yuwen Huang, Yang Peng, Weihao Liang, Xin He, Chen Chen, Jiayong Li, Wengen Zhu, Fang-fei Wei, Yugang Dong, Chen Liu, Yuzhong Wu

**Affiliations:** 1 Department of Cardiology, the First Affiliated Hospital of Sun Yat-Sen University, Guangzhou, 510080 People’s Republic of China; 2 NHC Key Laboratory of Assisted Circulation (Sun Yat-Sen University), Guangzhou, 510080 People’s Republic of China; 3 Department of Radiology, the First Affiliated Hospital of Sun Yat-Sen University, Guangzhou, 510080 People’s Republic of China; 4 National-Guangdong Joint Engineering Laboratory for Diagnosis and Treatment of Vascular Diseases, Guangzhou, People’s Republic of China

**Keywords:** Epicardial adipose tissue, Biomarkers, Myocardial strain, Heart failure, Mediation effect

## Abstract

**Background:**

Epicardial adipose tissue (EAT) has been suggested to exert deleterious effects on myocardium and cardiovascular disease (CVD) consequence. We evaluated the associations of EAT thickness with adverse outcomes and its potential mediators in the community.

**Methods:**

Participants without heart failure (HF) who had undergone cardiac magnetic resonance (CMR) to measure EAT thickness over the right ventricular free wall from the Framingham Heart Study were included. The correlation of EAT thickness with 85 circulating biomarkers and cardiometric parameters was assessed in linear regression models. The occurrence of HF, atrial fibrillation, coronary heart disease (CHD), and other adverse events was tracked since CMR was implemented. Their associations with EAT thickness and the mediators were evaluated using Cox regression and causal mediation analysis.

**Results:**

Of 1554 participants, 53.0% were females. Mean age, body mass index, and EAT thickness were 63.3 years, 28.1 kg/m^2^, and 9.8 mm, respectively. After fully adjusting, EAT thickness positively correlated with CRP, LEP, GDF15, MMP8, MMP9, ORM1, ANGPTL3, and SERPINE1 and negatively correlated with N-terminal pro-B-type natriuretic peptide (NT-proBNP), IGFBP1, IGFBP2, AGER, CNTN1, and MCAM. Increasing EAT thickness was associated with smaller left ventricular end-diastolic dimension, thicker left ventricular wall thickness, and worse global longitudinal strain (GLS). During a median follow-up of 12.7 years, 101 incident HF occurred. Per 1-standard deviation increment of EAT thickness was associated with a higher risk of HF (adjusted hazard ratio [HR] 1.43, 95% confidence interval [CI] 1.19–1.72, *P* < 0.001) and the composite outcome consisting of myocardial infarction, ischemic stroke, HF, and death from CVD (adjusted HR [95% CI], 1.23 [1.07–1.40], *P* = 0.003). Mediation effect in the association between thicker EAT and higher risk of HF was observed with NT-proBNP (HR [95% CI], 0.95 [0.92–0.98], *P* = 0.011) and GLS (HR [95% CI], 1.04 [1.01–1.07], *P* = 0.032).

**Conclusions:**

EAT thickness was correlated with inflammation and fibrosis-related circulating biomarkers, cardiac concentric change, myocardial strain impairment, incident HF risk, and overall CVD risk. NT-proBNP and GLS might partially mediate the effect of thickened EAT on the risk of HF. EAT could refine the assessment of CVD risk and become a new therapeutic target of cardiometabolic diseases.

**Trial registration:**

URL: https://clinicaltrials.gov. Identifier: NCT00005121.

**Supplementary Information:**

The online version contains supplementary material available at 10.1186/s12916-023-02836-4.

## Background

**Fig. 1:**
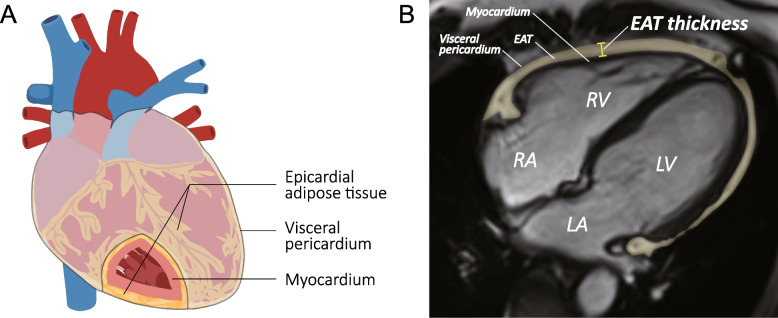
Localization and measurement of epicardial adipose tissue. **A** Epicardial adipose tissue lies between the myocardium and visceral pericardium. **B** Epicardial adipose tissue thickness was measured perpendicularly on the free wall of the right ventricle at the mid-ventricular level. EAT, epicardial adipose tissue; RV, right ventricle; RA, right atrium; LV, left ventricle; LA, left atrium

Epicardial adipose tissue (EAT) has gained much attention in recent years as its special anatomical location between the myocardium and visceral pericardium (Fig. 1A), and its functional orientation on the cardiovascular microenvironment, which is characterized by the paracrine or vasocrine secretion of pro-inflammatory and profibrotic cytokines [1, 2]. Increased EAT has been suggested to play a role in the development of coronary heart disease (CHD), atrial fibrillation (AF), and heart failure (HF) with preserved ejection fraction (HFpEF) [3]. Recent studies have linked coronary atherosclerosis and myocardial ischemia to EAT in patients with diabetes mellitus and in post-menopausal women [4, 5]. EAT also contributes to adverse myocardial remodeling after myocardial infarction [6]. In patients with CHD, EAT exhibits predicting ability for adverse cardiovascular events, especially for HFpEF [7, 8]. HFpEF patients have greater EAT thickness than HF with reduced ejection fraction (HFrEF) [9, 10]. And in HFpEF, the accumulation of EAT was reported to correlate with worse cardiac function, worse exercise capacity, and higher mortality [9–12]. Such effects of EAT are carried out by mechanical restraint, increased inflammation, autonomic dysregulation, impaired energy utilization, and so on [3, 13]. Current studies remain unclear as to the cardiovascular consequence and underlying mechanism of EAT thickening in the general population. The biochemical and myocardial relevancy of EAT and their contribution to the risk of adverse cardiovascular outcomes need to be explored. We aim to investigate the circulating biomarkers and cardiometric parameters that correlate with EAT thickness, evaluate the associations of EAT thickness with the risk of adverse cardiovascular outcomes, and assess whether these markers mediate the development of adverse outcomes in a community population.

## Methods

### Study design and participants

This study included community participants sampled from Framingham, MA, in the Framingham Heart Study (FHS) to conduct a longitudinal analysis. Data access was acquired from the National Center for Biotechnology Information of the USA. FHS is a long-term, multigenerational study designed to investigate constitutional and environmental factors influencing the development of cardiovascular disease (CVD) in men and women [14]. The cardiac magnetic resonance (CMR) which assessed EAT thickness was performed on participants in the offspring cohort during their examinations 7 and 8 in 2002 ~ 2006. The offspring cohort consisted of offspring of the original Framingham cohort and spouses of the offspring. The participants with CMR data and free of HF were included in this study. The investigation conforms with the principles outlined in the *Declaration of Helsinki*. The study protocol was approved by the Medical Ethical Committee of the First Affiliated Hospital, Sun Yat-sen University.

### EAT thickness and covariates

The EAT thickness was acquired from non-contrast CMR scanning on a 1.5-T Philips scanner (Gyroscan ACS-NT, Philips Medical Systems, Best, the Netherlands) with a 5-element cardiac array coil. After a scout scan, end-expiratory breath-hold, ECG-gated cine steady-state free precession images were acquired in 2-chamber, 4-chamber, and contiguous short axis orientations. Imaging parameters included TR = 3.2 ms, TE = 1.6 ms, flip angle = 60°, field-of-view 400 mm, and matrix size 208 × 256. Slice thickness was 10 mm, and in-plane spatial resolution was 1.9 mm × 1.6 mm, with 30 to 40 ms temporal resolution. Image analysis was performed using the software of MEDIS Inc., LEIDEN (version 6.1 QT). In the horizontal long-axis end-diastolic view (4-chamber), EAT thickness was measured perpendicularly on the free wall of the right ventricle at the mid-ventricular level, which crossed the midpoint between tricuspid annulus and apex cordis (Fig. 1B) [15–17].

The age at CMR implementation was recorded and marked the assumed beginning of follow-up. Other baseline information, including body mass index, blood pressure, estimated glomerular filtration rate (eGFR), plasma lipids, and so on, were extracted from the data of examination 8. The determination of the history of diabetes mellitus, atrial fibrillation, or CHD was by the physician-administered medical history review. Dyslipidemia was defined as triglycerides ≥ 150 mg/dL or low-density lipoprotein cholesterol ≥ 135 mg/dL or high-density lipoprotein cholesterol < 40 mg/dL for men and < 50 mg/dL for women.

### Circulating biomarkers and cardiac measures

During the same study period with CMR, plasma samples of the participants were acquired, and echocardiography was conducted. Plasma samples were utilized to perform immunoassays of 85 circulating protein biomarkers of atherosclerosis and metabolic syndrome. The technology being implemented was the Luminex™ xMAP assay, an extension of the enzyme-linked immunosorbent assay performed with multiple analyte-specific capture antibodies bound to a set of fluorescent beads. An xMAP assay could simultaneously quantify up to 100 analytes at abundances as low as picograms per milliliter in multiple samples. For measurement of cardiac structure and function, most parameters including left ventricular end-diastolic dimension (LVEDD), left atrial internal dimension (LAID), left ventricular wall thickness (LVWT), and left ventricular ejection fraction (LVEF) were directly obtained from CMR, and global longitudinal strain (GLS) and mitral inflow velocity to early diastolic mitral annular velocity (*E*/*é*) were assessed by echocardiography in examination 8 [18]. GLS was derived from speckle tracking stain-based analyses performed on digitally recorded 2-dimensional images.

### Outcomes

The follow-up examinations were conducted every 4 to 6 years to track the occurrence of incident HF, AF, CHD, and other adverse events through December 2017. Participants were diagnosed as having CHD if there existed one of the following definite manifestations: myocardial infarction, coronary insufficiency, angina pectoris, or sudden or non-sudden death from CHD. The composite outcome major adverse cardiovascular event (MACE) was defined as the presence of myocardial infarction, ischemic stroke, HF, or death from CVD. The detailed diagnostic criteria of each event are listed in Additional file 1: Table S1. The participants’ medical records were evaluated using a 3-physician panel by the Framingham Endpoint Review Committee for adjudicating the outcomes.

### Statistical analysis

Baseline characteristics were summarized using frequencies and proportions for categorical variables and mean and standard deviation (SD) for continuous variables and compared by *χ* ^2^ tests and unpaired Student’s *t*-tests, respectively. The participants were stratified according to the median of EAT thickness, which was consistent with the cut-off value of previous research [19]. The associations of EAT thickness with circulating biomarkers and cardiac measures were assessed using linear regression models, while the associations with clinical outcomes including HF, AF, CHD, and MACE were evaluated using Cox proportional hazards models. Cumulative incidence curves of HF stratified by EAT thickness were plotted. When assessing the associations of EAT thickness with the risk of AF or CHD, the participants who presented with AF or CHD at baseline had been excluded. The levels of circulating biomarkers had been processed by logarithmic transformation (natural logarithms) prior to further analysis. The covariates adjusted in each model were sex, age, body mass index, systolic blood pressure, diastolic blood pressure, eGFR, diabetes mellitus, atrial fibrillation, coronary heart disease, and dyslipidemia. Then, we further conducted a causal mediation analysis to see if any of the circulating biomarkers or cardiac measures alters the association between EAT thickness and clinical outcomes. First, we screened out the circulating biomarkers or cardiac measures which simultaneously correlated with both the EAT thickness and the clinical outcomes. Next, the shortlisted indicators were put into causal mediation analysis provided by Huang YT and Yang HI [20], as instrumental variables, with EAT thickness as the independent variable and clinical outcomes as the dependent variable. The statistical analyses were performed using R software version 4.0.2 (R Foundation for Statistical Computing, Vienna, Austria). A two-tailed *P*-value of < 0.05 was considered statistically significant.

## Results

### Baseline characteristics

The population characteristics at CMR implementation are shown in Table 1. The total 1554 participants had a mean age of 63.3 years (ranging from 35 to 88 years), and 53.0% females, with a mean body mass index of 28.1 kg/m^2^. The mean EAT thickness was 9.8 mm. The prevalence of diabetes mellitus, AF, CHD, and dyslipidemia were 12%, 4.1%, 7.5%, and 69.2%, respectively. The averages of LVEF, GLS, and *E*/*é* were 67.3%, − 20.6%, and 7.0, respectively. Compared to the participants with EAT < 9 mm, those with EAT ≥ 9 mm were predominantly males and older and had larger body mass index, greater systolic blood pressure, faster heart rate, higher triglyceride, lower high-density lipoprotein cholesterol, and higher fasting blood glucose. Participants with EAT ≥ 9 mm had a higher prevalence of diabetes mellitus, CHD, and dyslipidemia and had larger left atrial and left ventricular chambers, thicker LVWT, worse GLS, and worse *E*/*é*, compared to those with EAT < 9 mm. There was no difference in LVEF regarding different EAT. Besides, 788 participants had evaluated baseline right ventricular structure. Compared to the participants with EAT < 9 mm, those with EAT ≥ 9 mm had larger right ventricular diameter and area (Additional file 1: Table S2).

**Table 1:** Baseline characteristics according to EAT thickness

	**Overall**	**EAT < 9 mm**	**EAT ≥ 9 mm**	***P***
	***N*** ** = 1554**	***N*** ** = 785**	***N*** ** = 769**	
EAT, mm	9.8 (6.0)	5.3 (2.6)	14.4 (4.8)	< 0.001
Female, *N*	824 (53.0)	482 (61.4)	342 (44.5)	< 0.001
Age, years	63.3 (8.9)	62.6 (9.0)	64.1 (8.8)	0.002
BMI, kg/m^2^	28.1 (5.1)	26.8 (4.9)	29.4 (5.0)	< 0.001
SBP, mmHg	128.0 (16.7)	126.5 (16.6)	129.4 (16.7)	0.001
DBP, mmHg	73.6 (9.9)	73.5 (9.4)	73.7 (10.3)	0.72
eGFR, mL/(mL/min/1.73m^2^)	79.2 (16.4)	79.5 (15.4)	78.9 (17.5)	0.44
Heart rate, bpm	61.9 (10.1)	61.1 (10.0)	62.7 (10.3)	0.002
Triglycerides, mg/dL	114.9 (61.7)	105.0 (54.6)	125.0 (66.8)	< 0.001
LDL-c, mg/dL	106.8 (31.1)	108.2 (29.2)	105.4 (32.9)	0.07
HDL-c, mg/dL	57.6 (17.8)	61.0 (17.9)	54.1 (17.0)	< 0.001
FBG, mg/dL	105.5 (22.6)	102.5 (19.4)	108.5 (25.2)	< 0.001
**Comorbidities**, *N*				
Diabetes mellitus	186 (12.0)	69 (8.8)	140 (18.2)	< 0.001
Atrial fibrillation	63 (4.1)	28 (3.6)	35 (4.6)	0.39
Coronary heart disease	116 (7.5)	45 (5.7)	71 (9.2)	0.011
Dyslipidemia	1076 (69.2)	491 (62.5)	585 (76.1)	< 0.001
**Medications**, *N*				
ACEI/ARB	473 (30.4)	182 (23.2)	291 (37.8)	< 0.001
Beta-blocker	378 (24.3)	174 (22.2)	204 (26.5)	0.06
CCB	184 (11.8)	78 (9.9)	106 (13.8)	0.023
Diuretic	293 (18.9)	127 (16.2)	166 (21.6)	0.008
Statin	559 (36.0)	253 (32.2)	306 (39.8)	0.002
Antiplatelet agent	634 (40.8)	316 (40.3)	318 (41.4)	0.70
Antidiabetic agent	115 (7.4)	37 (4.7)	78 (10.1)	< 0.001
**Cardiac measures**				
LVEDD, mm	50.6 (4.7)	50.0 (4.6)	51.1 (4.7)	< 0.001
LAID, mm^a^	30.2 (5.3)	28.8 (5.0)	31.6 (5.2)	< 0.001
LVWT (septal), mm	7.9 (1.4)	7.6 (1.3)	8.1 (1.4)	< 0.001
LVWT (inferior), mm	6.8 (1.3)	6.5 (1.2)	7.0 (1.3)	< 0.001
LVEF, %	67.3 (6.7)	67.2 (6.0)	67.5 (7.3)	0.37
GLS, %^a^	− 20.6 (3.3)	− 21.1 (3.2)	− 20.2 (3.4)	< 0.001
*E*/*é* ^a^	7.0 (2.2)	6.9 (2.1)	7.2 (2.3)	0.005

Presented as number (percentage) or mean (SD)

*Abbreviations*: *EAT* epicardial adipose tissue, *BMI* body mass index, *SBP* systolic blood pressure, *DBP* diastolic blood pressure, *eGFR* estimated glomerular filtration rate, *HDL-c* high-density lipoprotein cholesterol, *LDL-c* low-density lipoprotein cholesterol, *FBG* fasting blood glucose, *ACEI* angiotensin-converting enzyme inhibitor, *ARB* angiotensin receptor blocker, *CCB* calcium channel blockers, *LVEDD* left ventricular end-diastolic dimension, *LAID* left atrial internal dimension, *LVWT* left ventricular wall thickness, *LVEF* left ventricular ejection fraction, *GLS* global longitudinal strain

^a^Missing values existed in LAID, GLS, and *E*/*é* (56, 95, and 70, respectively)

### Associations of circulating biomarkers and cardiac measures with EAT thickness

As presented in Table 2, after adjusting for common risk factors, EAT thickness was significantly correlated with 14 biomarkers. A positive correlation was found with CRP, LEP, GDF15, MMP8, MMP9, ORM1, ANGPTL3, and SERPINE1 and a negative correlation was with N-terminal pro-B-type natriuretic peptide (NT-proBNP), IGFBP1, IGFBP2, AGER, CNTN1, and MCAM. The expressions of these 14 biomarkers have significant differences between participants with EAT < 9 mm and ≥ 9 mm (Additional file 1: Table S3). The associations of all 85 biomarkers with EAT thickness are shown in Additional file 1: Table S4. For cardiac structure and function, per 1-SD increment of EAT thickness was associated with smaller LVEDD (*β* − 0.23, standard error [SE] 0.11, *P* = 0.040), thicker LVWT (septal, *β* [SE], 0.07 [0.03], *P* = 0.029; inferior, *β* [SE], 0.09 [0.03],* P* = 0.002), and worse GLS (*β* [SE], 0.34 [0.09], *P* < 0.001).

**Table 2:** Associations of serum biomarkers and cardiac measures with EAT thickness

	***β*** ** (standard error)**			
	**Crude**	***P***	**Adjusted**^**a**^	***P***
**Biomarkers, log pg/mL**				
CRP	0.20 (0.03)	< 0.001	0.06 (0.03)	0.030
LEP	0.15 (0.03)	< 0.001	0.08 (0.02)	0.001
GDF15	0.05 (0.01)	< 0.001	0.02 (0.01)	0.039
MMP8	0.10 (0.02)	< 0.001	0.05 (0.02)	0.035
MMP9	0.06 (0.01)	< 0.001	0.03 (0.01)	0.036
ORM1	0.04 (0.01)	< 0.001	0.02 (0.01)	0.003
ANGPTL3	0.06 (0.01)	< 0.001	0.02 (0.01)	0.023
SERPINE1	0.12 (0.01)	< 0.001	0.04 (0.01)	0.001
NT-proBNP	− 0.11 (0.02)	< 0.001	− 0.09 (0.02)	< 0.001
IGFBP1	− 0.24 (0.02)	< 0.001	− 0.09 (0.02)	< 0.001
IGFBP2	− 0.09 (0.01)	< 0.001	− 0.04 (0.01)	0.005
AGER	− 0.07 (0.01)	< 0.001	− 0.04 (0.01)	< 0.001
CNTN1	− 0.04 (0.01)	< 0.001	− 0.02 (0.01)	0.010
MCAM	− 0.02 (0.01)	0.001	− 0.02 (0.01)	0.018
**Cardiac measures**				
LVEDD, mm	0.40 (0.12)	0.001	− 0.23 (0.11)	0.040
LAID, mm	1.46 (0.13)	< 0.001	0.19 (0.12)	0.10
LVWT (septal), mm	0.29 (0.03)	< 0.001	0.07 (0.03)	0.029
LVWT (inferior), mm	0.30 (0.03)	< 0.001	0.09 (0.03)	0.002
LVEF, %	0.26 (0.17)	0.12	0.36 (0.17)	0.06
GLS, %	0.63 (0.09)	< 0.001	0.34 (0.09)	< 0.001
*E*/*é*	0.13 (0.06)	0.025	0.02 (0.06)	0.69

Biomarker description: CRP, C-reactive protein; LEP, leptin; GDF15, growth differentiation factor 15; MMP8, matrix metallopeptidase 8; MMP9, matrix metallopeptidase 9; ORM1, orosomucoid 1; ANGPTL3, angiopoietin-like 3; SERPINE1, serpin family E member 1; NT-proBNP, N-terminal pro-B-type natriuretic peptide; IGFBP1, insulin-like growth factor-binding protein 1; IGFBP2, insulin-like growth factor-binding protein 2; AGER, advanced glycation end products; CNTN1, contactin 1; MCAM, melanoma cell adhesion molecule

*Abbreviations*: *LVEDD* left ventricular end-diastolic dimension, *LAID* left atrial internal dimension, *LVWT* left ventricular wall thickness, *LVEF* left ventricular ejection fraction, *GLS* global longitudinal strain, *E/é* mitral inflow velocity to early diastolic mitral annular velocity

^a^Adjusted for sex, age, body mass index, systolic blood pressure, diastolic blood pressure, estimated glomerular filtration rate, diabetes mellitus, atrial fibrillation, coronary heart disease, and dyslipidemia

### Association between EAT thickness and adverse cardiovascular outcomes

**Fig. 2:**
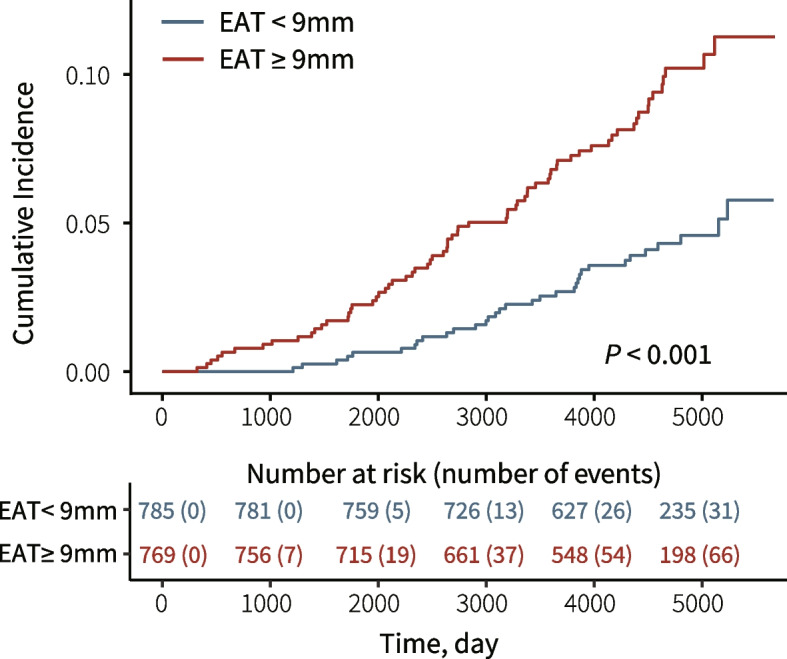
Cumulative incidence curves of heart failure stratified by EAT thickness. EAT, epicardial adipose tissue

The associations between EAT thickness and adverse outcomes including incident HF, AF, CHD, and the composite outcome MACE are reported in Table 3. There were 101 incident HF events that occurred during the follow-up of the median 12.7 years, and the incident rate was 0.5 (95% confidence interval [CI] 0.4–0.7) per 100 person-year. The population characteristics according to the occurrence of incident HF are shown in Additional file 1: Table S5. The baseline EAT thickness of the participants who suffered from incident HF in the follow-up has no difference between those with reduced LVEF and with normal or borderline LVEF. In both crude and multivariable models, per 1-SD increment in EAT thickness was correlated with a higher risk of HF (crude hazard ratio [HR] 1.57, 95% CI 1.35–1.83; adjusted HR [95% CI], 1.43 [1.19–1.72]; both *P* < 0.001). The HR of covariates in multivariable models are exhibited in Additional file 1: Table S6. Compared to EAT < 9 mm, participants with EAT ≥ 9 mm had distinctly more cumulative events of HF (Fig. 2), and their risk of HF was significantly higher (crude HR [95% CI], 2.28 [1.51–3.46], *P* < 0.001; adjusted HR [95% CI], 1.55 [1.01–2.38], *P* = 0.045). Per 1-SD increment of EAT thickness was also found to correlate with a higher risk of AF (HR [95% CI], 1.16 [1.01–1.32], *P* = 0.030) in crude analysis, but insignificant in the multivariable model. No association was observed between EAT thickness and the risk of CHD. In addition, per 1-SD increment of EAT thickness was associated with a higher risk of MACE (crude HR [95% CI], 1.42 [1.26–1.59], *P* < 0.001; adjusted HR [95% CI], 1.23 [1.07–1.40], *P* = 0.003).

**Table 3:** Associations between EAT thickness and adverse cardiovascular outcomes

		**Events/** ***N***	**Incidence, per 100 person-year**	**Hazard ratio (95% confidence interval)**			
				**Crude**	***P***	**Adjusted**^**a**^	***P***
**HF**	**Overall** ^b^	101/1554	0.5 (0.4–0.7)	1.57 (1.35–1.83)	< 0.001	1.43 (1.19–1.72)	< 0.001
	**EAT < 9 mm**	33/785	0.3 (0.2–0.5)	ref		ref	
	**EAT ≥ 9 mm**	68/769	0.8 (0.6–1.0)	2.28 (1.51–3.46)	< 0.001	1.55 (1.01–2.38)	0.045
**AF**	**Overall** ^b^	197/1491	1.2 (1.0–1.4)	1.16 (1.01–1.32)	0.030	0.95 (0.83–1.10)	0.53
	**EAT < 9 mm**	93/757	1.1 (0.9–1.3)	ref		ref	
	**EAT ≥ 9 mm**	104/734	1.3 (1.1–1.6)	1.22 (0.92–1.62)	0.16	0.81 (0.53–1.24)	0.34
**CHD**	**Overall** ^b^	95/1438	0.5 (0.4–0.7)	1.09 (0.90–1.33)	0.38	0.97 (0.78–1.20)	0.77
	**EAT < 9 mm**	49/740	0.5 (0.4–0.7)	ref		ref	
	**EAT ≥ 9 mm**	46/698	0.6 (0.4–0.7)	1.04 (0.70–1.56)	0.84	0.79 (0.52–1.21)	0.28
**MACE**	**Overall** ^b^	206/1554	1.1 (1.0–1.3)	1.42 (1.26–1.59)	< 0.001	1.23 (1.07–1.40)	0.003
	**EAT < 9 mm**	84/785	0.9 (0.7–1.1)	ref		ref	
	**EAT ≥ 9 mm**	122/769	1.4 (1.1–1.6)	1.59 (1.20–2.10)	0.001	1.11 (0.83–1.48)	0.49

*Abbreviations*: *EAT* epicardial adipose tissue, *HF* heart failure, *AF* atrial fibrillation, *CHD* coronary heart disease, *MACE* major adverse cardiovascular event

^a^Adjusted for sex, age, body mass index, systolic blood pressure, diastolic blood pressure, estimated glomerular filtration rate, diabetes mellitus, atrial fibrillation (not applicable for the outcome AF), coronary heart disease (not applicable for the outcome CHD), and dyslipidemia

^b^The hazard ratios corresponded to per 1-standard deviation increment of EAT thickness

### Mediation analysis

**Fig. 3:**
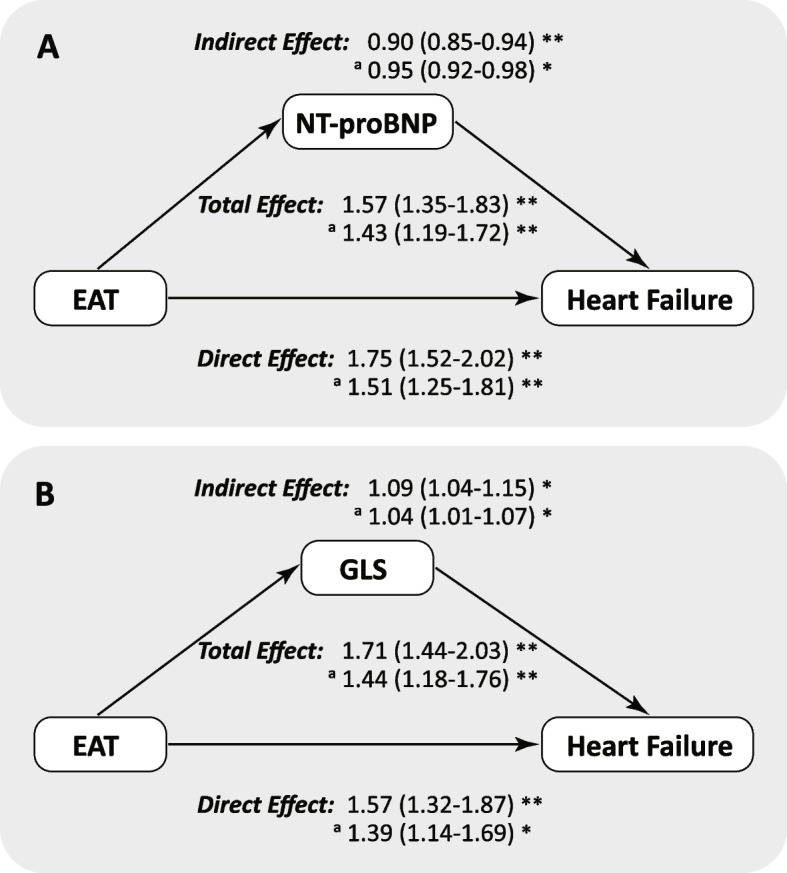
NT-proBNP and GLS mediate the effect of EAT on the risk of heart failure. **A** Mediation effect of NT-proBNP. **B** Mediation effect of GLS. The total, direct, and indirect effects are expressed by hazard ratios. EAT, epicardial adipose tissue; NT-proBNP, N-terminal pro-B-type natriuretic peptide; GLS, global longitudinal strain; ^*^ *P* < 0.05; ^**^ *P* < 0.001. ^a^Adjusted for sex, age, body mass index, systolic blood pressure, diastolic blood pressure, estimated glomerular filtration rate, diabetes mellitus, atrial fibrillation, coronary heart disease, and dyslipidemia

Precede to the mediation analysis, associations between the presupposed mediators (including biomarkers and cardiac measures) and the risk of HF were assessed. Of the 22 biomarkers and the cardiac measures (including LVEDD, LAID, LVWT, LVEF, GLS, and *E*/*é*) that were correlated with the risk of HF (Additional file 1: Table S7), CRP, GDF15, NT-proBNP, IGFBP2, LVEDD, LVWT, and GLS were detected to have a mediation effect for the association between EAT thickness and HF risk (Fig. 3 and Additional file 1: Table S8). After fully adjusting, NT-proBNP and GLS still showed a robust mediation effect (Fig. 3). The direct effect (HR [95% CI], 1.51 [1.25–1.81], *P* < 0.001) between thicker EAT and higher risk of HF was weakened by the indirect effect through NT-proBNP (HR [95% CI], 0.95 [0.92–0.98], *P* = 0.011), resulting in a milder total effect (HR [95% CI], 1.43 [1.19–1.72], *P* < 0.001). Meanwhile, the direct effect (HR [95% CI], 1.39 [1.14–1.69], *P* = 0.001) between thicker EAT and higher risk of HF was strengthened by the indirect effect of GLS (HR [95% CI], 1.04 [1.01–1.07], *P* = 0.032), resulting in a greater total effect (HR [95% CI], 1.44 [1.18–1.76], *P* < 0.001).

## Discussion

In a large community-based sample, we observed that (i) EAT thickness was correlated with 14 circulating biomarkers including NT-proBNP, as well as the dimension, wall thickness, and myocardial strain of left ventricle; (ii) thickened EAT was associated with a higher risk of incident HF and the outcome composited by myocardial infarction, ischemic stroke, HF, or death from CVD; and (iii) NT-proBNP and GLS had a mediation effect for the association between EAT thickness and HF risk.

As a well-recognized source of pro-inflammatory cytokines affecting the biochemical processes involved in cardiac dysfunction, EAT releases leptin, IL-6, IL-1β, TNF-α, MCP-1, ANGPTL2, visfatin, adiponectin, resistin, omentin, and other bioactive molecules [1, 21]. Some of those, such as IL-1β, IL-6, TNF-α, and MCP-1, were associated with myocardial collagen deposition and fibrosis [21, 22]. Biomarkers related to myocardial injury such as creatine kinase-MB, troponin T, and glycated hemoglobin were reported to be positively correlated with epicardial fat volume in patients with HFpEF or HF with mid-range ejection fraction [9, 23]. Our study identified additional CVD and metabolic syndrome-related markers that were associated with EAT thickness. Consistent with the prior studies, NT-proBNP showed an inverse correlation with EAT [9, 13]. Given that NT-proBNP is usually lower in HFpEF than in HFrEF patients for any given LV filling pressure, thicker EAT might tend to endorse the occurrence of HFpEF [24]. Besides, our results indicated that IGFBP1 and IGFBP2 negatively correlated with EAT thickness. IGFBP1 and IGFBP2 alter the interaction of insulin-like growth factors with their cell surface receptors, while insulin-like growth factor 1 receptor signaling is a key pathway that governs EAT formation by redirecting the fate of Wt1 + lineage cells [2].

As a risk factor in the general population, EAT was found to correlate with the risk of incident HF and the composite outcome of MACE in our study. In prior cross-sectional analyses, peri-atrial and peri-coronary EAT were related to the development of AF and coronary atherosclerosis, respectively [4, 5, 25]. In a longitudinal study, EAT volume was also positively related to the duration and recurrence of AF [26]. The impact of EAT on the risk of AF might result from the electroanatomical dysregulation and wall fibrosis caused by the EAT surrounding the left atrium [3]. From the above, we could tell that EAT is not equally distributed throughout the heart and its function is regionally dependent. EAT in different locations has diverse transcriptome and proteome, which influence the near heart structures in different ways [27–29]. Since the EAT measured in this study was adjacent to the right ventricle, it is explainable that we failed to prove the relevancy between EAT thickness and the risk of incident CHD or AF. In the non-HF population, increased EAT has been proven to link with HFpEF development and a higher overall risk of cardiovascular adverse events in patients with CHD [7, 8]. Herein, we added that EAT thickness also correlated with the risk of HF in the community. Moreover, increase right ventricle localized EAT was associated with a higher risk of frequent ventricular premature beats [30]. Collectively, the promotion on both myocardial remodeling and arrhythmogenesis of EAT was suggested.

Patients with HFpEF displayed thicker EAT than non-HF controls and HF patients with reduced or mid-range ejection fraction [9, 10, 23]. And actually, HFrEF patients tended to have thinner EAT than controls [9, 31, 32]. This stressed the importance of EAT in the development of HFpEF. In HFpEF, increased EAT thickness is associated with worse GLS of both LA and LV, higher right-sided filling pressures, worse peak oxygen consumption and peripheral extraction, and higher mortality and HF hospitalization [9–12, 33]. These associations regarding cardiac function, hemodynamic status, and survival were weaker or absent, even opposite in HFrEF [9, 10, 33]. Moreover, greater EAT mass in HFpEF was associated with higher cardiac extracellular volume, which indicated more distinct myocardial fibrosis [33]. Among obese HFpEF patients with increased EAT, more concentric left ventricular remodeling, greater elevation in cardiac filling pressures, and worse exercise capacity were observed compared with non-obese HFpEF patients and non-HF controls [13].

EAT can affect the risk and severity of HFpEF via several mechanisms, such as increased mechanical restraint, inflammation, fibrosis, and autonomic dysregulation. The pro-inflammatory secretome of EAT is involved in endothelial function, extracellular matrix remodeling, coagulation, immune signaling, and apoptosis [29]. EAT/miRNA axis was also involved as a new pathological mechanism, explaining the adverse myocardial remodeling occurring after MI [6]. The gene expression pattern of peri-atrial EAT was related to cardiac muscle contraction and intracellular calcium signaling pathway [27]. Moreover, there exists a vicious circle created by the promotion of systemic inflammation on the accumulation of EAT [34]. Among the biochemical alterations caused by thickened EAT, the reduction of natriuretic peptide is likely to play a critical role in the development of HFpEF. Attenuated activation of natriuretic peptide signaling decreases the secretion of adiponectin and promotes adipose tissue inflammation and cardiac fibrosis [34]. Natriuretic peptide signaling also contributes to the changes in EAT metabolism in cardiac cachexia [35]. In the present study, a lower level of BNP which related to thicker EAT mediated a decreasing risk of HF in the general population, and this might be attributed to the accelerated breakdown of natriuretic peptides in people at risk of HFpEF [36].

Increased EAT thickness changes myocardial energy metabolism. In substrate utilization, it causes increased oxygen consumption, impaired oxygen use, and increased dependence on fatty acid oxidation [13]. It may also increase the ATP transfer rate through creatine kinase to compensate for depleted energy stores, exhibited by a saturated ATP shuttling in cardiomyocytes, and thus contributes to a reduction in the ability to enhance energy utilization under stress conditions [37]. In addition, the right ventricle might be particularly affected by EAT on account that approximately 75% of EAT resides over the right ventricle [38]. The pericardial restraint caused by EAT accumulation might facilitate right ventricular–arterial uncoupling, resulting in enhanced systolic ventricular interdependence and increased extravascular lung water, especially in HFpEF [13, 19, 39].

EAT provides an unconventional perspective on the pathophysiology of HF and other CVDs. Our findings supported the idea that EAT accumulation might alter the myocardial microenvironment and ultimately promote the fibrosis and stiffening of the myocardium, leading to myocardial remodeling and HF. Since the relationships between EAT thickness and prognosis in patients with HFpEF versus HFrEF were almost opposing in many situations, further studies are needed to specify the divergent roles of EAT in the pathophysiology of HF with different LVEF stratification [9, 10, 33]. Overall, indexes of EAT might solely, or joined with other metabolic parameters, become a tool for assessing the CVD risk in the general population. Since EAT can also be assessed by echocardiography with the most ready availability and lowest cost, a relatively ideal cost-effectiveness could be realized. In the meantime, EAT is a modifiable cardiovascular risk factor that has been shown to be regulated by lifestyle changes and pharmacological interventions. Thus, EAT could become a new therapeutic target of cardiometabolic diseases. Meta-analysis revealed that significant EAT reduction occurred with diet control and bariatric surgery but not with exercise, and the response to lifestyle changes was associated with higher secretion of adiponectin and leptin and decreased expression of pro-inflammatory adipokines [40]. Statins, metformin, and sodium-glucose cotransporter 2 inhibitors have been shown to reduce the quantity of EAT and to ameliorate its local pro-inflammatory effects and the influence on metabolic dysregulation [34, 41]. Still, it needs to be further investigated whether these therapies indeed improve outcomes through targeting at EAT and their heterogeneity in HF patients with different ejection fractions.

### Strengths and limitations

To our knowledge, this is the first study to demonstrate a significant prognostic role of EAT in the general population, rather than in patients with specific CVD. And we identified the mediators NT-proBNP and GLS between EAT and the risk of HF, suggesting the potential biochemical and mechanistic links between them. However, due to the retrospective nature of the present analysis, causality cannot be deduced. Besides, there were a limited number of incident HF cases, which might influence the estimates from the regression models. CMR was used in this study, providing greater precision and accuracy to quantify EAT thickness than echocardiography, whereas late gadolinium enhancement and evaluation of incidental intramyocardial fatty infiltration were not available, which could provide more prognostic information. In addition, the current study only assessed the thickness, not the volume, mass, or quality of EAT, and measured EAT only over the right ventricular free wall. Other limitations included that the participants were not multi-racial, resulting in the inadequacy of representativeness. At last, we failed to distinguish the classification of LVEF of all the participants who developed HF in the follow-up; thus, we could not determine whether the long-term risk related to thickening EAT is more predisposed to a specific type of HF.

## Conclusions

In the general population, EAT thickness was correlated with inflammation and fibrosis-related circulating biomarkers, cardiac concentric change, myocardial strain impairment, incident HF risk, and overall CVD risk. NT-proBNP and GLS might partially mediate the effect of thickened EAT on the risk of HF. EAT could refine the assessment of CVD risk and become a new therapeutic target of cardiometabolic diseases.

## Supplementary Information

**Additional file 1: Table S1. **Detailed diagnostic criteria of study outcomes. **Table S2. **Baseline right ventricle indexes according to EAT thickness. **Table S3. **The differences of the shortlisted 14 circulating biomarkers according to EAT thickness. **Table S4. **The associations of all 85 circulating biomarkers with EAT thickness. **Table S5. **Baseline characteristics according to occurrence of incident heart failure and LVEF stratification in those with incident heart failure. **Table S6.** The hazard ratio of EAT and adjusted factors in univariate and multivariate models which assessed their association with incident heart failure. **Table S7. **Associations of circulating biomarkers and cardiac measures with incident heart failure. **Table S8. **Mediation analyses of circulating biomarkers and cardiac measures in the association of EAT with the risk of heart failure.

## Data Availability

The dataset of the Framingham Heart Study is available via reasonable request to the National Center for Biotechnology Information of the USA.

## Abbreviations

AF: Atrial fibrillation
CHD: Coronary heart disease
CI: Confidence interval
CMR: Cardiac magnetic resonance
CVD: Cardiovascular disease
*E*/*é*: Mitral inflow velocity to early diastolic mitral annular velocity
EAT: Epicardial adipose tissue
eGFR: Estimated glomerular filtration rate
FHS: Framingham Heart Study
GLS: Global longitudinal strain
HF: Heart failure
HFpEF: Heart failure with preserved ejection fraction
HFrEF: Heart failure with reduced ejection fraction
HR: Hazard ratio
LAID: Left atrial internal dimension
LVEDD: Left ventricular end-diastolic dimension
LVEF: Left ventricular ejection fraction
LVWT: Left ventricular wall thickness
MACE: Major adverse cardiovascular event
NT-proBNP: N-terminal pro-B-type natriuretic peptide
SD: Standard deviation
SE: Standard error
