# Supplementary material for: Association between epicardial adipose tissue and incident heart failure mediating by alteration of natriuretic peptide and myocardial strain
Source: BMC Med. 2023 Mar 29;21:117. doi: 10.1186/s12916-023-02836-4 (PMC10053458; doi:10.1186/s12916-023-02836-4)
Supplement: Supplementary file 1 — Additional file 1: Table S1. Detailed diagnostic criteria of study outcomes. Table S2. Baseline right ventricle indexes according to EAT thickness. Table S3. The differences of the shortlisted 14 circulating biomarkers according to EAT thickness. Table S4. The associations of all 85 circulating biomarkers with EAT thickness. Table S5. Baseline characteristics according to occurrence of incident heart failure and LVEF stratification in those with incident heart failure. Table S6. The hazard ratio of EAT and adjusted factors in univariate and multivariate models which assessed their association with incident heart failure. Table S7. Associations of circulating biomarkers and cardiac measures with incident heart failure. Table S8. Mediation analyses of circulating biomarkers and cardiac measures in the association of EAT with the risk of heart failure. [file 12916_2023_2836_MOESM1_ESM.docx]

**Table S1. Detailed diagnostic criteria of study outcomes.**

| **Heart failure (HF)** | A definite diagnosis of heart failure requires that a minimum of two major or one major and two minor criteria be present concurrently. The presence of other conditions capable of producing the symptoms and signs are considered in evaluating the findings.  Major Criteria:   1. Paroxysmal nocturnal dyspnea or orthopnea; 2. Distended neck veins (in other than the supine position); 3. Rales; 4. Increasing heart size by x-ray; 5. Acute pulmonary edema on chest x-ray; 6. Ventricular S(3) gallop;   7) Increased venous pressure > 16 cm H20;  8) Hepatojugular reflux;  9) Pulmonary edema, visceral congestion, cardiomegaly shown on autopsy;  10) Weight loss on CHF Rx: 10 lbs.**/**5days.  Minor criteria:   1. Bilateral ankle edema; 2. Night cough; 3. Dyspnea on ordinary exertion; 4. Hepatomegaly; 5. Pleural effusion by x-ray; 6. Decrease in vital capacity by one-third from maximum record; 7. Tachycardia (120 beats per minute or more); 8. Pulmonary vascular engorgement on chest x-ray. |
| --- | --- |
| **Coronary heart disease (CHD)** | Subjects are diagnosed as having developed coronary heart disease (CHD) if upon review of the case a panel of three investigators (the Framingham Endpoint Review Committee) agrees on one of the following definite manifestations of CHD: myocardial infarction, coronary insufficiency, angina pectoris, sudden death from CHD, non-sudden death from CHD.  **The various manifestations of CHD are:**  *Angina Pectoris*  Brief recurrent chest discomfort of up to 15 minutes duration, precipitated by exertion or emotion and relieved by rest or by nitroglycerine is regarded as angina pectoris (AP) if two physicians interviewing the subject at a Framingham clinic visit or the Framingham Endpoint Review Committee, upon review of medical records, agree that this condition was definitely present. This diagnosis is based solely on evaluation of subjective manifestations. Abnormality of the resting or exercise electrocardiogram is not required for this diagnosis.  *Myocardial Infarction*  Recent or acute myocardial infarction (MI) is designated when there were at least two of three findings:   1. symptoms indicative of ischemia; 2. changes in biomarkers of myocardial necrosis; 3. serial changes in the electrocardiograms indicating the evolution of an infarction, including the loss of initial QRS potentials (that is, development of “pathologic” Q-waves of 0.04 second duration or greater).   An old or remote myocardial infarction is considered to be present when the electrocardiogram shows a stable pattern including a pathologic Q-wave of 0.04 second or greater or loss of initial QRS potential R-wave in those leads in which this would not be expected to occur. Also, an interim unrecognized MI is indicated when changes from a previous tracing show development of loss of R-wave potential or appearance of pathologic Q-waves not otherwise explained, in persons in whom neither the patient nor his physician considered the possibility of MI. If the patient was asymptomatic for chest pain or upper abdominal pain during the interval at which the unrecognized MI occurred, the event is classified as silent, unrecognized. More weight is given to this finding if a T-wave abnormality is also associated with Q-wave abnormality.  An autopsy report showing an acute, new, or recent infarction of the myocardium is accepted as evidence of an incident myocardial infarction. Because it is not possible to date an old infarction found on autopsy, such evidence is not used in the clinical diagnosis of a new event, unless there was an interim clinical event suspected of being an infarction.  *Coronary Insufficiency*  The coronary insufficiency syndrome is designated when a history of prolonged ischemic chest pain (> 15 minutes duration) was accompanied by transient ischemic S-T segment and T-wave abnormality in the electrocardiographic tracing but not accompanied by development of Q-wave abnormality or by serum enzyme changes characteristic of myocardial necrosis.  *Coronary Heart Disease Death*  Death from coronary heart disease is diagnosed as either sudden or nonsudden. |
| **Ischemic Stroke** | The diagnosis of cerebrovascular disease is based on the occurrence of a clinically evident stroke documented by clinical records reviewed by at least two neurologists. Stroke is defined as the sudden or rapid onset of a focal neurologic deficit persisting for greater than 24 hours.  A diagnosis of cerebral embolism is made when an established source for embolus including atrial fibrillation, rheumatic heart disease with mitral stenosis, recent myocardial infarction, bacterial endocarditis or other known source is determined. A clinical course consistent with embolic infarction or evidence of other systemic embolism may be present. Symptoms are usually rapid with maximal severity at onset.  Antherothrombotic brain infarction is defined as the sudden onset of a focal neurologic deficit lasting longer than 24 hours, in the absence of:  1) known source of embolism (atrial fibrillation, rheumatic heart disease with mitral stenosis, myocardial infarction within preceding six months, bacterial endocarditis);  2) intracranial hemorrhage (intracerebral or subarachnoid);  3) known hypercoagulable states;  4) other disease processes causing focal neurologic deficits (brain tumor, subdural hematoma, hypoglycemia).  Confirmatory imaging supports the diagnosis. |

**Table S2. Baseline right ventricle indexes according to EAT thickness.**

|  | **Overall** | **EAT< 9mm** | **EAT≥ 9mm** | ***P*** |
| --- | --- | --- | --- | --- |
|  | **N=788** | **N=434** | **N=354** |  |
| RVEDA | 17.8 (4.4) | 17.6 (4.4) | 18.2 (4.3) | 0.044 |
| RVESA | 7.9 (2.8) | 7.7 (2.7) | 8.2 (2.8) | 0.009 |
| RVFAC | 0.6 (0.1) | 0.6 (0.1) | 0.6 (0.1) | 0.014 |
| RVD1 | 3.5 (0.6) | 3.4 (0.6) | 3.5 (0.6) | 0.53 |
| RVD2 | 2.8 (0.6) | 2.8 (0.6) | 2.9 (0.6) | 0.039 |
| RVD3 | 7.4 (0.9) | 7.4 (0.9) | 7.5 (0.9) | 0.048 |

These right ventricle indexes were measured by echocardiography in apical 4-chamber view. Presented as mean (SD). RVEDA, right ventricular end-diastolic area; RVESA, right ventricular end-systolic area; RVFAC, right ventricular fractional area change; RVD1, right ventricular diameter measured at the basal level; RVD2, right ventricular diameter measured at the mid-ventricular level; RVD3, right ventricular diameter measured from base to apex.

**Table S3. The differences of the shortlisted 14 circulating biomarkers according to EAT thickness.**

| **Markers, log pg/mL** | **Overall** | **EAT< 9mm** | **EAT≥ 9mm** | ***P*** |
| --- | --- | --- | --- | --- |
|  | **N=1554** | **N=785** | **N=769** |  |
| CRP | 13.8 (1.2) | 13.6 (1.2) | 13.9 (1.2) | <0.001 |
| GDF15 | 6.4 (0.4) | 6.3 (0.4) | 6.4 (0.4) | <0.001 |
| MMP8 | 5.8 (0.8) | 5.7 (0.9) | 5.9 (0.8) | <0.001 |
| MMP9 | 10.8 (0.5) | 10.8 (0.5) | 10.9 (0.5) | 0.001 |
| NT-proBNP | 5.1 (1.0) | 5.2 (1.0) | 5.0 (1.0) | <0.001 |
| AGER | 8.1 (0.3) | 8.2 (0.3) | 8.1 (0.3) | <0.001 |
| LEP | 8.4 (1.2) | 8.3 (1.2) | 8.5 (1.2) | <0.001 |
| ORM1 | 20.3 (0.3) | 20.3 (0.3) | 20.4 (0.3) | <0.001 |
| ANGPTL3 | 10.1 (0.4) | 10.0 (0.4) | 10.2 (0.4) | <0.001 |
| SERPINE1 | 9.8 (0.5) | 9.7 (0.5) | 9.9 (0.5) | <0.001 |
| CNTN1 | 10.7 (0.2) | 10.8 (0.2) | 10.7 (0.2) | <0.001 |
| IGFBP1 | 9.0 (1.0) | 9.2 (0.9) | 8.8 (1.0) | <0.001 |
| IGFBP2 | 16.1 (0.6) | 16.2 (0.6) | 16.0 (0.6) | <0.001 |
| MCAM | 11.9 (0.2) | 12.0 (0.2) | 11.9 (0.2) | 0.008 |

Presented as mean and standard deviation. Abbreviations: EAT, epicardial adipose tissue.

Biomarker description: CRP, C-reactive protein; LEP, leptin; GDF15, growth differentiation factor 15; MMP8, matrix metallopeptidase 8; MMP9, matrix metallopeptidase 9; ORM1, orosomucoid 1; ANGPTL3, angiopoietin-like 3; SERPINE1, serpin family E member 1; NT-proBNP, N-terminal pro-B-type natriuretic peptide; IGFBP1, insulin-like growth factor-binding protein 1; IGFBP2, insulin-like growth factor-binding protein 2; AGER, advanced glycation endproducts; CNTN1, contactin 1; MCAM, melanoma cell adhesion molecule.

**Table S4. The associations of all 85 circulating biomarkers with EAT thickness.**

| **UniProt accession** | **Gene** | **Description** |  | **β (Standard Error)** | | | |  |
| --- | --- | --- | --- | --- | --- | --- | --- | --- |
|  |  |  | **Crude** | | ***P*** | **Adjusted *** | ***P*** | |
| [P02760](https://www.uniprot.org/uniprot/P02760) | AMBP | Alpha-1-microglobulin | 0.01 (0.01) | | 0.54 | -0.00 (0.01) | 0.78 | |
| [O14639](https://www.uniprot.org/uniprot/O14639) | ABLIM1 | Actin binding LIM protein 1 | -0.00 (0.01) | | 0.69 | -0.01 (0.01) | 0.46 | |
| [Q13444](https://www.uniprot.org/uniprot/Q13444) | ADAM15 | Disintegrin and metalloproteinase domain-containing protein 15 | -0.01 (0.02) | | 0.53 | -0.03 (0.02) | 0.28 | |
| [P00746](https://www.uniprot.org/uniprot/P00746) | CFD | Adipsin | 0.03 (0.01) | | <0.001 | 0.00 (0.01) | 0.76 | |
| [P35318](https://www.uniprot.org/uniprot/P35318) | ADM | Adrenomedullin | 0.02 (0.01) | | <0.001 | 0.00 (0.01) | 0.62 | |
| [P02763](https://www.uniprot.org/uniprot/P02763) | ORM1 | Orosomucoid 1, Alpha-1-acid glycoprotein | 0.04 (0.01) | | <0.001 | 0.02 (0.01) | 0.003 | |
| [P22557](https://www.uniprot.org/uniprot/P22557) | ALAS2 | Aminolevulinate, delta-, synthase 2 | -0.01 (0.01) | | 0.06 | -0.02 (0.01) | 0.026 | |
| [P04083](https://www.uniprot.org/uniprot/P04083) | ANXA1 | Annexin A1 | -0.01 (0.01) | | 0.25 | -0.01 (0.01) | 0.36 | |
| [Q9Y5C1](https://www.uniprot.org/uniprot/Q9Y5C1) | ANGPTL3 | Angiopoietin-like 3 | 0.06 (0.01) | | <0.001 | 0.02 (0.01) | 0.023 | |
| [P02647](https://www.uniprot.org/uniprot/P02647) | APOA1 | Apolipoprotein-A1 | -0.01 (0.01) | | 0.31 | 0.00 (0.01) | 0.82 | |
| [P04114](https://www.uniprot.org/uniprot/P04114) | APOB-100 | Apolipoprotein-B100 | -0.00 (0.01) | | 0.70 | -0.01 (0.01) | 0.17 | |
| [P04114](https://www.uniprot.org/uniprot/P04114) | APOB | Apolipoprotein B | 0.03 (0.01) | | <0.001 | 0.01 (0.01) | 0.06 | |
| [P61769](https://www.uniprot.org/uniprot/P61769) | B2M | Beta-2-microglobulin | 0.02 (0.01) | | 0.012 | -0.00 (0.01) | 0.72 | |
| [P06276](https://www.uniprot.org/uniprot/P06276) | BCHE | Butyrylcholine esterase | 0.02 (0.01) | | <0.001 | 0.01 (0.01) | 0.20 | |
| [P02760](https://www.uniprot.org/uniprot/P02760) | BIKUNIN | Ambp-bikunin | 0.00 (0.01) | | 0.80 | 0.01 (0.01) | 0.24 | |
| [P16860](https://www.uniprot.org/uniprot/P16860) | NPPB | NT-proBNP (N-terminal prohormone of brain natriuretic peptide) | -0.11 (0.02) | | <0.001 | -0.09 (0.02) | <0.001 | |
| [P06681](https://www.uniprot.org/uniprot/P06681) | C2 | Complement C2 | 0.01 (0.00) | | 0.22 | -0.00 (0.00) | 0.99 | |
| [P08571](https://www.uniprot.org/uniprot/P08571) | CD14 | Monocyte differentiation antigen CD14 | 0.00 (0.01) | | 0.90 | 0.00 (0.01) | 0.42 | |
| [Q86VB7](https://www.uniprot.org/uniprot/Q86VB7) | CD163 | Scavenger receptor cysteine-rich type 1 protein (M130) | 0.04 (0.01) | | 0.003 | 0.01 (0.01) | 0.59 | |
| [P29965](https://www.uniprot.org/uniprot/P29965) | CD40L | Soluble CD40 ligand | 0.02 (0.01) | | 0.15 | 0.01 (0.01) | 0.29 | |
| [P13591](https://www.uniprot.org/uniprot/P13591) | NCAM1 | Neural cell adhesion molecule (CD56) | -0.01 (0.01) | | 0.11 | -0.02 (0.01) | 0.002 | |
| [O43866](https://www.uniprot.org/uniprot/O43866) | CD5L | CD5 antigen-like | 0.02 (0.01) | | 0.09 | -0.01 (0.01) | 0.32 | |
| [P55290](https://www.uniprot.org/uniprot/P55290) | CDH13 | Cadherin-13 | -0.01 (0.03) | | 0.84 | -0.02 (0.03) | 0.52 | |
| [P00450](https://www.uniprot.org/uniprot/P00450) | CP | Ceruloplasmin | -0.02 (0.01) | | 0.001 | 0.00 (0.01) | 0.93 | |
| [Q12860](https://www.uniprot.org/uniprot/Q12860) | CNTN1 | Contactin 1 | -0.04 (0.01) | | <0.001 | -0.02 (0.01) | 0.01 | |
| [P39060](https://www.uniprot.org/uniprot/P39060) | COL18A1 | Collagen, type XVIII, alpha 1 | 0.01 (0.01) | | 0.14 | 0.00 (0.01) | 0.98 | |
| [P02741](https://www.uniprot.org/uniprot/P02741) | CRP | C-reactive protein | 0.20 (0.03) | | <0.001 | 0.06 (0.03) | 0.03 | |
| [P32927](https://www.uniprot.org/uniprot/P32927) | CSF2RB | Colony stimulating factor 2 receptor, beta, low-affinity (granulocyte-macrophage) | 0.00 (0.02) | | 0.83 | 0.01 (0.02) | 0.61 | |
| [Q9H2A7](https://www.uniprot.org/uniprot/Q9H2A7) | CXCL16 | Chemokine (C-X-C motif) ligand 16 | 0.00 (0.01) | | 0.89 | -0.00 (0.01) | 0.99 | |
| [P01034](https://www.uniprot.org/uniprot/P01034) | CST3 | Cystatin-C | 0.02 (0.01) | | 0.001 | 0.00 (0.01) | 0.58 | |
| [P27487](https://www.uniprot.org/uniprot/P27487) | DPP4 | Dipeptidyl-peptidase 4 | -0.01 (0.01) | | 0.41 | 0.01 (0.01) | 0.59 | |
| [Q12805](https://www.uniprot.org/uniprot/Q12805) | EFEMP1 | EGF containing fibulin-like extracellular matrix protein 1 | 0.00 (0.01) | | 0.54 | -0.01 (0.01) | 0.38 | |
| [Q8TC29](https://www.uniprot.org/uniprot/Q8TC29) | ENKUR | Enkurin | 0.00 (0.00) | | 0.56 | 0.00 (0.00) | 0.36 | |
| [P02671](https://www.uniprot.org/uniprot/P02671) | FBG | Fibrinogen | 0.02 (0.01) | | 0.002 | 0.00 (0.01) | 0.99 | |
| [Q9GZV9](https://www.uniprot.org/uniprot/Q9GZV9) | FGF23 | Fibroblast growth factor 23 | -0.02 (0.02) | | 0.28 | -0.00 (0.02) | 0.84 | |
| [P36888](https://www.uniprot.org/uniprot/P36888) | FLT3 | FMS-related tyrosine kinase 3 | -0.06 (0.04) | | 0.07 | -0.02 (0.04) | 0.53 | |
| [P04406](https://www.uniprot.org/uniprot/P04406) | GAPDH | Glyceraldehyde 3-phosphate dehydrogenase | 0.01 (0.01) | | 0.54 | 0.00 (0.01) | 0.93 | |
| [Q99988](https://www.uniprot.org/uniprot/Q99988) | GDF15 | Growth differentiation factor 15 | 0.05 (0.01) | | <0.001 | 0.02 (0.01) | 0.039 | |
| [P16109](https://www.uniprot.org/uniprot/P16109) | SELP | Granule membrane protein 140 (P-selectin) | 0.03 (0.01) | | 0.002 | 0.00 (0.01) | 0.65 | |
| [P40197](https://www.uniprot.org/uniprot/P40197) | GP5 | Glycoprotein V (platelet) | -0.01 (0.01) | | 0.26 | -0.00 (0.01) | 0.94 | |
| [P28799](https://www.uniprot.org/uniprot/P28799) | GRN | Granulin | 0.01 (0.01) | | 0.32 | 0.01 (0.01) | 0.13 | |
| [P10144](https://www.uniprot.org/uniprot/P10144) | GZMB | Granzyme B | -0.04 (0.03) | | 0.09 | -0.03 (0.03) | 0.26 | |
| [P02790](https://www.uniprot.org/uniprot/P02790) | HPX | Hemopexin | 0.01 (0.00) | | 0.011 | 0.01 (0.01) | 0.24 | |
| [P22304](https://www.uniprot.org/uniprot/P22304) | IDS | Iduronate 2-sulfatase | 0.02 (0.02) | | 0.12 | 0.02 (0.02) | 0.18 | |
| [P05019](https://www.uniprot.org/uniprot/P05019) | IGF1 | Insulin-like growth factor 1 | -0.07 (0.01) | | <0.001 | -0.02 (0.02) | 0.20 | |
| [P08069](https://www.uniprot.org/uniprot/P08069) | IGF1R | Insulin like growth factor 1 receptor | -0.00 (0.01) | | 0.94 | 0.00 (0.01) | 0.92 | |
| [P17936](https://www.uniprot.org/uniprot/P17936) | IGFBP3 | Insulin-like growth factor-binding protein 3 | -0.01 (0.01) | | 0.31 | -0.00 (0.01) | 0.68 | |
| [P08833](https://www.uniprot.org/uniprot/P08833) | IGFBP1 | Insulin-like growth factor-binding protein 1 | -0.24 (0.02) | | <0.001 | -0.09 (0.02) | <0.001 | |
| [P18065](https://www.uniprot.org/uniprot/P18065) | IGFBP2 | Insulin-like growth factor binding protein 2 | -0.09 (0.01) | | <0.001 | -0.04 (0.01) | 0.005 | |
| [P05231](https://www.uniprot.org/uniprot/P05231) | IL6 | Interleukin 6 | -0.00 (0.00) | | 0.91 | -0.00 (0.00) | 0.64 | |
| [P03952](https://www.uniprot.org/uniprot/P03952) | KLKB1 | Plasma kallikrein | 0.00 (0.01) | | 0.79 | 0.01 (0.01) | 0.13 | |
| [P01130](https://www.uniprot.org/uniprot/P01130) | LDLR | Low density lipoprotein receptor | -0.01 (0.02) | | 0.82 | -0.02 (0.03) | 0.47 | |
| [P41159](https://www.uniprot.org/uniprot/P41159) | LEP | Leptin | 0.15 (0.03) | | <0.001 | 0.08 (0.02) | 0.001 | |
| [P08519](https://www.uniprot.org/uniprot/P08519) | LPA | Lipoprotein(a) | 0.01 (0.04) | | 0.74 | 0.03 (0.04) | 0.49 | |
| [P43121](https://www.uniprot.org/uniprot/P43121) | MCAM | Melanoma cell adhesion molecule | -0.02 (0.01) | | 0.001 | -0.02 (0.01) | 0.018 | |
| [P13500](https://www.uniprot.org/uniprot/P13500) | CCL2 | Monocyte chemotactic molecule 1 | 0.02 (0.01) | | 0.012 | -0.00 (0.01) | 0.95 | |
| [P14780](https://www.uniprot.org/uniprot/P14780) | MMP9 | Matrix metallopeptidase 9 | 0.05 (0.01) | | <0.001 | 0.03 (0.01) | 0.036 | |
| [P22894](https://www.uniprot.org/uniprot/P22894) | MMP8 | Matrix metallopeptidase 8 | 0.10 (0.02) | | <0.001 | 0.05 (0.02) | 0.035 | |
| [P05164](https://www.uniprot.org/uniprot/P05164) | MPO | Myeloperoxidase | 0.02 (0.01) | | 0.11 | 0.00 (0.01) | 0.80 | |
| [P02144](https://www.uniprot.org/uniprot/P02144) | MB | Myoglobin | 0.04 (0.01) | | <0.001 | -0.01 (0.01) | 0.33 | |
| [P46531](https://www.uniprot.org/uniprot/P46531) | NOTCH1 | Translocation associated notch homolog | 0.01 (0.01) | | 0.44 | -0.01 (0.01) | 0.47 | |
| [Q92823](https://www.uniprot.org/uniprot/Q92823) | NRCAM | Neuronal cell adhesion molecule | -0.02 (0.03) | | 0.48 | -0.03 (0.03) | 0.36 | |
| [P02818](https://www.uniprot.org/uniprot/P02818) | BGLAP | Osteocalcin | -0.02 (0.02) | | 0.12 | -0.00 (0.02) | 0.83 | |
| [P05121](https://www.uniprot.org/uniprot/P05121) | SERPINE1 | Plasminogen activator inhibitor 1 | 0.12 (0.01) | | <0.001 | 0.04 (0.01) | 0.001 | |
| [P02689](https://www.uniprot.org/uniprot/P02689) | PMP2 | Myelin P2 protein | -0.01 (0.02) | | 0.43 | -0.01 (0.02) | 0.48 | |
| [P00491](https://www.uniprot.org/uniprot/P00491) | PNP | Purine nucleoside phosphorylase | -0.01 (0.01) | | 0.34 | -0.00 (0.01) | 0.82 | |
| [P27169](https://www.uniprot.org/uniprot/P27169) | PON1 | Serum paraoxonase/arylesterase 1 | -0.04 (0.01) | | 0.001 | -0.01 (0.01) | 0.34 | |
| [P02775](https://www.uniprot.org/uniprot/P02775) | PPBP | Pro-platelet basic protein | 0.01 (0.02) | | 0.58 | 0.01 (0.03) | 0.81 | |
| [Q9UK55](https://www.uniprot.org/uniprot/Q9UK55) | SERPINA10 | Protein Z-dependent protease inhibitor | -0.01 (0.01) | | 0.48 | -0.01 (0.01) | 0.52 | |
| [P05451](https://www.uniprot.org/uniprot/P05451) | REG1A | Lithostathine-1-alpha | 0.01 (0.01) | | 0.40 | 0.01 (0.01) | 0.39 | |
| [Q9HD89](https://www.uniprot.org/uniprot/Q9HD89) | RETN | Resistin | 0.00 (0.01) | | 0.87 | -0.01 (0.01) | 0.19 | |
| [Q01196](https://www.uniprot.org/uniprot/Q01196) | RUNX1 | Runt-related transcription factor 1 | -0.02 (0.01) | | 0.06 | -0.01 (0.01) | 0.28 | |
| [P0DJI8](https://www.uniprot.org/uniprot/P0DJI8) | SAA1 | Serum amyloid A1 | 0.06 (0.02) | | 0.017 | -0.01 (0.02) | 0.68 | |
| [P48061](https://www.uniprot.org/uniprot/P48061) | CXCL12 | Stromal cell-derived factor 1 | -0.01 (0.01) | | 0.26 | 0.01 (0.01) | 0.27 | |
| [P40189](https://www.uniprot.org/uniprot/P40189) | IL6ST | Interleukin-6 receptor beta | 0.01 (0.01) | | 0.26 | 0.00 (0.01) | 0.95 | |
| [P05362](https://www.uniprot.org/uniprot/P05362) | ICAM1 | Intercellular adhesion molecule 1 | 0.02 (0.01) | | 0.002 | 0.01 (0.01) | 0.33 | |
| [Q15109](https://www.uniprot.org/uniprot/Q15109) | AGER | Advanced glycation endproducts | -0.07 (0.01) | | <0.001 | -0.04 (0.01) | <0.001 | |
| [P05452](https://www.uniprot.org/uniprot/P05452) | CLEC3B | Tetranectin | -0.01 (0.00) | | 0.16 | 0.00 (0.00) | 0.92 | |
| [P07996](https://www.uniprot.org/uniprot/P07996) | THBS1 | Thrombospondin-1 | -0.00 (0.03) | | 0.88 | -0.01 (0.04) | 0.84 | |
| [P01033](https://www.uniprot.org/uniprot/P01033) | TIMP1 | Tissue inhibitor of metalloproteinases 1 | 0.02 (0.01) | | <0.001 | 0.00 (0.01) | 0.68 | |
| [P19429](https://www.uniprot.org/uniprot/P19429) | TNNI3 | Troponin | 0.00 (0.01) | | 0.41 | 0.01 (0.01) | 0.26 | |
| [Q99576](https://www.uniprot.org/uniprot/Q99576) | TSC22D3 | TSC22D3 domain family, member 3 | -0.01 (0.01) | | 0.27 | -0.01 (0.01) | 0.50 | |
| [P08493](https://www.uniprot.org/uniprot/P08493) | MGP | Uncarboxylated matrix gla protein | 0.04 (0.01) | | <0.001 | 0.01 (0.01) | 0.21 | |
| [P15692](https://www.uniprot.org/uniprot/P15692) | VEGF | Vascular endothelial growth factor | 0.03 (0.02) | | 0.10 | 0.02 (0.02) | 0.27 | |
| [Q9H4A3](https://www.uniprot.org/uniprot/Q9H4A3) | WNK1 | WNK lysine deficient protein kinase 1 | -0.11 (0.06) | | 0.07 | -0.10 (0.07) | 0.18 | |

***** Adjusted for sex, age, body mass index, systolic blood pressure, diastolic blood pressure, estimated glomerular filtration rate, diabetes mellitus, atrial fibrillation, coronary heart disease, dyslipidemia.

**Table S5. Baseline characteristics according to occurrence of incident heart failure and LVEF stratification in those with incident heart failure.**

|  | **Without incident HF** | **With incident HF** | ***P*** | **HF with reduced LVEF** † | **HF with normal or borderline LVEF** † | ***P*** |
| --- | --- | --- | --- | --- | --- | --- |
|  | **N=1453** | **N=101** |  | **N=29** | **N=50** |  |
| EAT, mm | 9.6 (5.7) | 12.8 (8.1) | <0.001 | 12.8 (8.8) | 12.8 (6.7) | 0.97 |
| Female, N | 779 (53.6) | 45 (44.6) | 0.10 | 9 (31.0) | 23 (46.0) | 0.29 |
| Age, years | 62.8 (8.8) | 71.2 (7.4) | <0.001 | 70.3 (8.7) | 71.8 (6.7) | 0.40 |
| BMI, kg/m^2^ | 28.0 (5.1) | 30.0 (5.9) | <0.001 | 29.1 (5.8) | 31.5 (5.4) | 0.07 |
| SBP, mmHg | 127.6 (16.4) | 133.7 (18.9) | <0.001 | 134.9 (22.5) | 134.4 (17.7) | 0.91 |
| DBP, mmHg | 74.0 (9.6) | 67.7 (11.7) | <0.001 | 69.4 (12.0) | 67.4 (12.8) | 0.49 |
| eGFR, mL/(min*1.73m^2^) | 80.0 (15.7) | 67.6 (21.8) | <0.001 | 70.0 (18.5) | 63.3 (24.4) | 0.21 |
| Heart rate, bpm | 61.8 (10.2) | 63.3 (9.6) | 0.16 | 64.7 (13.1) | 62.7 (8.1) | 0.42 |
| Triglycerides, mg/dL | 114.5 (61.5) | 121.4 (65.0) | 0.28 | 138.0 (98.0) | 113.6 (44.1) | 0.13 |
| LDL-c, mg/dL | 107.8 (31.1) | 93.2 (28.6) | <0.001 | 89.3 (27.3) | 91.6 (28.1) | 0.72 |
| HDL-c, mg/dL | 58.0 (17.9) | 51.4 (16.0) | <0.001 | 46.0 (17.3) | 52.9 (12.8) | 0.049 |
| FBG, mg/dL | 105.1 (21.9) | 111.7 (30.9) | 0.005 | 112.2 (19.5) | 111.2 (26.1) | 0.86 |
| **Comorbidities** |  |  |  |  |  |  |
| Diabetes mellitus, N | 156 (10.7) | 30 (29.7) | <0.001 | 11 (37.9) | 18 (36.0) | 1.00 |
| Atrial fibrillation, N | 50 (3.4) | 13 (12.9) | <0.001 | 4 (13.8) | 5 (10.0) | 0.89 |
| Coronary heart disease, N | 92 (6.3) | 24 (23.8) | <0.001 | 9 (31.0) | 11 (22.0) | 0.53 |
| Dyslipidemia, N | 1000 (68.8) | 76 (75.2) | 0.21 | 26 (89.7) | 37 (74.0) | 0.17 |
| **Cardiac measures** **at baseline** |  |  |  |  |  |  |
| LVEDD, mm | 50.5 (4.6) | 52.1 (5.7) | 0.001 | 54.2 (5.4) | 51.4 (5.7) | 0.031 |
| LAID, mm ***** | 29.9 (5.0) | 34.5 (6.5) | <0.001 | 35.4 (7.2) | 34.9 (6.7) | 0.77 |
| LVWT (Septal), mm | 7.8 (1.4) | 8.5 (1.5) | <0.001 | 8.9 (1.4) | 8.4 (1.5) | 0.17 |
| LVWT (Inferior), mm | 6.7 (1.2) | 7.4 (1.4) | <0.001 | 8.0 (1.5) | 7.2 (1.1) | 0.006 |
| LVEF, % | 67.4 (6.2) | 66.5 (11.7) | 0.18 | 59.9 (13.1) | 70.1 (9.8) | <0.001 |
| GLS, % ***** | -20.8 (3.2) | -19.1 (4.0) | <0.001 | -17.3 (4.0) | -19.8 (3.7) | 0.008 |
| E/e' ***** | 6.9 (2.1) | 8.8 (3.3) | <0.001 | 8.2 (2.9) | 9.3 (3.4) | 0.19 |

Presented as number (percentage) or mean (SD).

Abbreviations: EAT, epicardial adipose tissue; HF, heart failure; BMI, body mass index; SBP, systolic blood pressure; DBP, diastolic blood pressure; eGFR, estimated glomerular filtration rate; HDL-c, high density lipoprotein cholesterol; LDL-c, low density lipoprotein cholesterol; FBG, fasting blood glucose; LVEDD, left ventricular end-diastolic dimension; LAID, left atrial internal dimension; LVWT, left ventricular wall thickness; LVEF, left ventricular ejection fraction; GLS, global longitudinal strain.

***** Missing values existed in LAID, GLS and E/e' (56, 95 and 70, respectively, in total study population).

† HF with reduced LVEF was defined as LVEF≤45%. HF with normal or borderline LVEF was defined as LVEF>45%. Of note, there were 22 incident HF events without information of LVEF.

**Table S6. The hazard ratio of EAT and adjusted factors in univariate and multivariate models which assessed their association with incident heart failure.**

|  |  | **Hazard Ratio (95% Confidence Interval)** | | | |  | |
| --- | --- | --- | --- | --- | --- | --- | --- |
|  | **Univariate model** | | ***P*** | **Multivariate model** | ***P*** | |  |
| EAT (per 1-SD) | 1.57 (1.35-1.83) | | <0.001 | 1.43 (1.19-1.72) | <0.001 | |  |
| Female sex | 0.65 (0.44-0.97) | | 0.034 | 0.72 (0.48-1.09) | 0.12 | |  |
| Age | 1.13 (1.10-1.16) | | <0.001 | 1.09 (1.06-1.12) | <0.001 | |  |
| SBP | 1.02 (1.01-1.03) | | <0.001 | 1.01 (1.00-1.03) | 0.023 | |  |
| DBP | 0.93 (0.91-0.95) | | <0.001 | 0.96 (0.93-0.98) | <0.001 | |  |
| BMI | 1.07 (1.03-1.10) | | <0.001 | 1.07 (1.03-1.11) | <0.001 | |  |
| eGFR | 0.96 (0.95-0.97) | | <0.001 | 0.98 (0.97-0.99) | 0.002 | |  |
| Diabetes mellitus | 3.74 (2.46-5.67) | | <0.001 | 1.46 (0.93-2.29) | 0.10 | |  |
| Atrial fibrillation | 4.58 (2.56-8.21) | | <0.001 | 1.66 (0.88-3.12) | 0.12 | |  |
| Coronary heart disease | 4.78 (3.02-7.57) | | <0.001 | 2.44 (1.48-4.04) | 0.001 | |  |
| Dyslipidemia | 1.40 (0.89-2.20) | | 0.14 | 0.76 (0.48-1.22) | 0.26 | |  |

**Table S7. Associations of** **circulating biomarkers and cardiac measures with incident heart failure.**

|  |  | **Hazard Ratio (95% Confidence Interval)** | | | | | |  |
| --- | --- | --- | --- | --- | --- | --- | --- | --- |
|  | **Crude** | | | | ***P*** | **Adjusted*** | ***P*** | |
| **Biomarkers, log pg/mL** | | |  | |  |  |  | |
| CRP | 1.56 (1.32-1.84) | | | | <0.001 | 1.32 (1.10-1.57) | 0.002 | |
| CST3 | 20.90 (10.45-41.81) | | | | <0.001 | 3.00 (1.05-8.58) | 0.04 | |
| GDF15 | 12.51 (8.32-18.79) | | | | <0.001 | 5.41 (3.00-9.77) | <0.001 | |
| MPO | 2.63 (1.52-4.54) | | | | 0.001 | 1.88 (1.11-3.16) | 0.018 | |
| NT-proBNP | 2.35 (1.90-2.90) | | | | <0.001 | 1.56 (1.24-1.96) | <0.001 | |
| MB | 3.35 (2.12-5.29) | | | | <0.001 | 1.87 (1.01-3.44) | 0.046 | |
| COL18A1 | 7.79 (3.65-16.60) | | | | <0.001 | 2.53 (1.15-5.59) | 0.021 | |
| ADM | 23.74 (10.30-54.72) | | | | <0.001 | 4.51 (1.58-12.90) | 0.005 | |
| CD14 | 5.93 (2.48-14.18) | | | | <0.001 | 2.87 (1.14-7.20) | 0.025 | |
| CFD | 12.81 (6.99-23.48) | | | | <0.001 | 2.46 (1.18-5.16) | 0.017 | |
| APOA1 | 0.44 (0.26-0.72) | | | | 0.001 | 0.59 (0.36-0.97) | 0.037 | |
| SAA1 | 1.60 (1.32-1.93) | | | | <0.001 | 1.29 (1.05-1.60) | 0.017 | |
| GRN | 5.14 (2.42-10.90) | | | | <0.001 | 3.52 (1.55-8.00) | 0.003 | |
| AMBP | 3.82 (2.48-5.88) | | | | <0.001 | 1.70 (1.02-2.82) | 0.040 | |
| EFEMP1 | 11.87 (7.42-19.01) | | | | <0.001 | 4.12 (2.11-8.04) | <0.001 | |
| IGFBP2 | 2.12 (1.49-3.01) | | | | <0.001 | 1.81 (1.21-2.70) | 0.004 | |
| FBG | 8.82 (3.95-19.70) | | | | <0.001 | 3.32 (1.46-7.55) | 0.004 | |
| IGF1R | 1.33 (1.05-1.69) | | | | 0.017 | 1.29 (1.02-1.65) | 0.036 | |
| BIKUNIN | 3.39 (1.56-7.39) | | | | 0.002 | 2.32 (1.00-5.35) | 0.049 | |
| NOTCH1 | 2.27 (1.43-3.60) | | | | 0.001 | 1.80 (1.06-3.06) | 0.029 | |
| TSC22D3 | 0.53 (0.24-1.14) | | | | 0.10 | 0.43 (0.20-0.92) | 0.03 | |
| MGP | 4.68 (2.86-7.65) | | | | <0.001 | 2.12 (1.29-3.50) | 0.003 | |
| **Cardiac measures** | | | |  |  |  |  | |
| LVEDD, mm | 1.08 (1.04-1.12) | | | | <0.001 | 1.07 (1.02-1.12) | 0.003 | |
| LAID, mm | 1.17 (1.13-1.21) | | | | <0.001 | 1.08 (1.04-1.13) | <0.001 | |
| LVWT (Septal), mm | 1.36 (1.20-1.54) | | | | <0.001 | 1.24 (1.07-1.44) | 0.004 | |
| LVWT (Inferior), mm | 1.44 (1.26-1.64) | | | | <0.001 | 1.34 (1.13-1.60) | 0.001 | |
| LVEF, % | 0.98 (0.95-1.01) | | | | 0.16 | 0.96 (0.94-0.99) | 0.005 | |
| GLS, % | 1.18 (1.11-1.26) | | | | <0.001 | 1.13 (1.06-1.20) | <0.001 | |
| E/e' | 1.26 (1.20-1.33) | | | | <0.001 | 1.19 (1.11-1.27) | <0.001 | |

***** Adjusted for sex, age, body mass index, systolic blood pressure, diastolic blood pressure, estimated glomerular filtration rate, diabetes mellitus, atrial fibrillation, coronary heart disease, dyslipidemia.

Abbreviations: LVEDD, left ventricular end-diastolic dimension; LAID, left atrial internal dimension; LVWT, left ventricular wall thickness; LVEF, left ventricular ejection fraction; GLS, global longitudinal strain.

**Table S8. Mediation analyses of circulating biomarkers and cardiac measures in the association of EAT with the risk of heart failure.**

|  |  | |  | **Hazard Ratio (95% Confidence Interval)** | | | |  |
| --- | --- | --- | --- | --- | --- | --- | --- | --- |
|  |  | | **Crude** | | ***P*** | **Adjusted*** | ***P*** | |
| **Biomarkers, log pg/mL** | |  |  | |  |  |  | |
| **CRP** | **TE** | | 1.65 (1.40-1.93) | | <0.001 | 1.43 (1.19-1.73) | <0.001 | |
|  | **DE** | | 1.52 (1.30-1.78) | | <0.001 | 1.41 (1.17-1.69) | <0.001 | |
|  | **IE** | | 1.08 (1.04-1.14) | | 0.001 | 1.02 (1.00-1.04) | 0.11 | |
| **GDF15** | **TE** | | 1.53 (1.33-1.76) | | <0.001 | 1.41 (1.18-1.68) | <0.001 | |
|  | **DE** | | 1.34 (1.18-1.53) | | <0.001 | 1.37 (1.15-1.63) | <0.001 | |
|  | **IE** | | 1.14 (1.08-1.20) | | <0.001 | 1.03 (1.00-1.06) | 0.08 | |
| **IGFBP2** | **TE** | | 1.52 (1.31-1.75) | | <0.001 | 1.39 (1.16-1.68) | <0.001 | |
|  | **DE** | | 1.65 (1.43-1.90) | | <0.001 | 1.43 (1.19-1.72) | <0.001 | |
|  | **IE** | | 0.92 (0.88-0.95) | | <0.001 | 0.97 (0.95-0.99) | 0.07 | |
| **Cardiac measures** |  | |  | |  |  |  | |
| **LVEDD, mm** | **TE** | | 1.65 (1.40-1.95) | | <0.001 | 1.42 (1.17-1.72) | <0.001 | |
|  | **DE** | | 1.61 (1.37-1.90) | | <0.001 | 1.45 (1.19-1.75) | <0.001 | |
|  | **IE** | | 1.03 (1.01-1.05) | | 0.036 | 0.98 (0.96-1.00) | 0.12 | |
| **LVWT (Septal), mm** | **TE** | | 1.55 (1.33-1.80) | | <0.001 | 1.39 (1.16-1.67) | <0.001 | |
|  | **DE** | | 1.45 (1.24-1.69) | | <0.001 | 1.37 (1.14-1.65) | <0.001 | |
|  | **IE** | | 1.07 (1.03-1.12) | | 0.004 | 1.01 (1.00-1.04) | 0.14 | |
| **LVWT (Inferior), mm** | **TE** | | 1.56 (1.34-1.82) | | <0.001 | 1.41 (1.17-1.70) | <0.001 | |
|  | **DE** | | 1.44 (1.23-1.69) | | <0.001 | 1.38 (1.14-1.66) | <0.001 | |
|  | **IE** | | 1.09 (1.04-1.14) | | 0.001 | 1.02 (1.00-1.05) | 0.06 | |
| **LVEF, %** | **TE** | | 1.59 (1.36-1.85) | | <0.001 | 1.40 (1.15-1.69) | <0.001 | |
|  | **DE** | | 1.60 (1.37-1.86) | | <0.001 | 1.41 (1.17-1.71) | <0.001 | |
|  | **IE** | | 0.99 (0.98-1.00) | | 0.29 | 0.99 (0.97-1.00) | 0.14 | |

***** Adjusted for sex, age, body mass index, systolic blood pressure, diastolic blood pressure, estimated glomerular filtration rate, diabetes mellitus, atrial fibrillation, coronary heart disease, dyslipidemia.

Abbreviations: LVEDD, left ventricular end-diastolic dimension; LVWT, left ventricular wall thickness; LVEF, left ventricular ejection fraction; TE, total effect; DE, direct effect; IE, indirect effect.
